# Effect of oral Colchicine on Peripapillary retinal nerve fiber layer thickness in patients with familial Mediterranean fever

**DOI:** 10.1186/s12886-018-0698-1

**Published:** 2018-02-05

**Authors:** Burak Tanyıldız, Mehmet Engin Tezcan, Baran Kandemir, Nesrin Tutaş Günaydın, Eren Göktaş, Aise Tangılntız, Aysu Karatay Arsan

**Affiliations:** 1 Dr. Lutfi Kirdar Kartal Education and Research Hospital, Department of Ophthalmology, Semsi Denizer Caddesi, E-5, 34890 Kartal Istanbul, Turkey; 2 Dr. Lutfi Kirdar Kartal Education and Research Hospital, Department of Rheumatology, Istanbul, Turkey; 3 Dr. Lutfi Kirdar Kartal Education and Research Hospital, Department of Family Medicine, Istanbul, Turkey

**Keywords:** Colchicine, Familial Mediterranean fever, Optic coherence tomography, Peripapillary retinal nerve fiber layer thickness

## Abstract

**Background:**

The purpose of this study is to investigate whether oral colchicine has an effect on peripapillary retinal nerve fiber layer (pRNFL) thickness of familial Mediterranean fever (FMF) patients.

**Methods:**

We conducted a cross sectional study by comparing pRNFL thickness of FMF patients on colchicine (treated group), newly diagnosed colchicine naïve FMF patients (untreated group) and healthy controls. The study included 66 FMF patients and 32 healthy control subjects. Treated FMF patients were grouped according to colchicine use, duration of use and dosage. pRNFL thickness of the patients and controls were measured by using optical coherence tomography and the measurements were compared.

**Results:**

No statistically significant difference was found between the pRNFL thickness in untreated group, treated group and the healthy control group (all *p* > 0.05). No statistically significant difference was found between pRNFL thickness in the healthy control group and FMF patients grouped according to duration or dosage of colchicine use (all p > 0.05).

**Conclusions:**

According to our study, FMF and oral colchicine use had no statistically significant effect on pRNFL thickness.

## Background

Familial Mediterranean fever (FMF) is a hereditary autoinflammatory disorder characterized by recurrent episodes of fever and polyserositis or arthritis [1–3]. The attacks are usually self-limiting and are mainly seen in people of Mediterranean origin such as Turks, Arabs, Armenians and Sephardic Jews [4, 5].

Increased serum levels of IL-1β and TNF-α in FMF patients have been reported both during and between the attacks [6, 7]. IL-1β and TNF-α cytokines have also been reported to cause optic neuropathy and retinal ganglion cell degeneration in certain animal studies [8, 9]. These facts suggest that the peripapillary retinal nerve fiber layer (pRNFL) and ganglion cell-inner plexiform layer (GCIPL) thicknesses can be affected in FMF patients as reported in some studies [10, 11]. Another study has emphasized that increased inflammation may also alter the choroidal thickness by causing vascular problems [12] Alim et al. [13] have also mentioned a possible effect of oral colchicine on pRNFL, GCIPL and choroidal thickness. However, measurements were taken while the patients were receiving colchicine treatment in these studies.

Colchicine is the gold standard in the treatment of FMF. It reduces the frequency and intensity of acute febrile attacks and inhibits the development of amyloidosis [14]. Some animal studies have shown that intravitreal administration of colchicine damages retinal ganglion cells and amacrine cells [15, 16]. In this study we aimed to evaluate the possible effects of oral colchicine on pRNFL thickness. The primary purpose of this study was to compare the pRNFL thickness in FMF patients already using oral colchicine and newly-diagnosed FMF patients scheduled to be started oral colchicine. The secondary purpose was to determine whether pRNFL values in FMF patients are affected by colchicine usage duration, the colchicine dose.

## Methods

We first obtained approval from the local ethics committee and conducted the study according to the tenets of the Declaration of Helsinki. The study was explained in detail to the participants and a signed informed consent form obtained. The FMF patients had been referred from the Rheumatology Department of Dr. Lutfi Kirdar Kartal Education and Research Hospital for eye examinations between April 2015 and February 2016. All FMF patients fulfilled the Tel-Hashomer criteria and all patients were evaluated by the same rheumatologist (M.E.T.) [17]. We also had a control group consisting of age- and sex-matched healthy volunteers who had come to our outpatient ophthalmology clinic for a routine eye examination.

We then performed a complete ophthalmologic evaluation, including best-corrected visual acuity (BCVA), anterior segment biomicroscopy, intraocular pressure measurement by Goldmann applanation tonometry, and dilated fundus examination for all subjects. All included participants’ best corrected visual acuity was 20/20 or better and intraocular pressure was < 20 mmHg. We excluded any patient with refractive error greater than +/− 1.00 diopter on cycloplegia, history of trauma or previous ocular surgery, cornea or lens opacity, uveitis, detectable posterior vitreous detachment, vitreopapillary traction, glaucoma (defined as vertical enlargement of the optic cup, > 0.2 cup/disc between the two eyes, localized loss of rim), retinal disorders, optic disc abnormalities, amblyopia, or neurological disorders that could affect the visual field.

FMF patients were divided into two groups as those who were diagnosed recently and were scheduled to start colchicine and those who were already on colchicine treatment. The patients using colchicine were classified according to the duration of colchicine treatment as 6–24 months and over 24 months. Patients on colchicine treatment less than 6 months were excluded from the study.

Newly diagnosed FMF patients (untreated group) were referred from the rheumatology clinic in the first visit. Then, we started colchicine treatment on the same day after ophthalmologic examinations and pRNFL measurements. The patient group was divided into two groups according to the colchicine dose as less than 1.5 mg/day and 1.5 mg/day and more.

To measure the pRNFL thickness, we used spectral-domain (SD) - Optical coherence tomography (Optos SD-OCT, Scotland, UK) with the fast pRNFL scan protocol. This protocol conducts three consecutive 360° circular scans of an area 3.4 mm in diameter with the optic disc at the center and automatically provides the pRNFL thicknesses for all four quadrants (superior, nasal, inferior, and temporal) together with the mean value. An internal fixation target was used in the OCT device for all the scans. Only the right eyes were assessed. These values were collected and compared between the groups.

### Statistical analysis

SPSS Version 17.0 (SPSS Inc., Chicago, IL, USA) was used for statistical analyses. The Kolmogorov-Smirnov test was initially used to determine whether the data were distributed normally. The groups did not show a normal distribution. We compared continuous variables between the groups by Kruskal-Wallis test. The results are presented as arithmetic mean ± standard deviation and a *p* value < 0.05 was considered statistically significant.

## Results

We enrolled a total of 76 FMF patients and 32 healthy control subjects (10 males and 22 females). Five patients had myopia over − 1.00 diopter and 4 patients had hypermetropia over + 1.00 diopter. One patient had Behcet’s disease and FMF together and hypopyon uveitis was seen during the follow-up. The hypopyon shifted freely with changes in head position. Diffuse fern-like leakage was seen from peripheral retinal vessels on fundus fluorescein angiography. This patient was later excluded from the study because of Behcet’s disease uveitis. We therefore excluded a total of 10 FMF patients from the study.

**Fig. 1:**
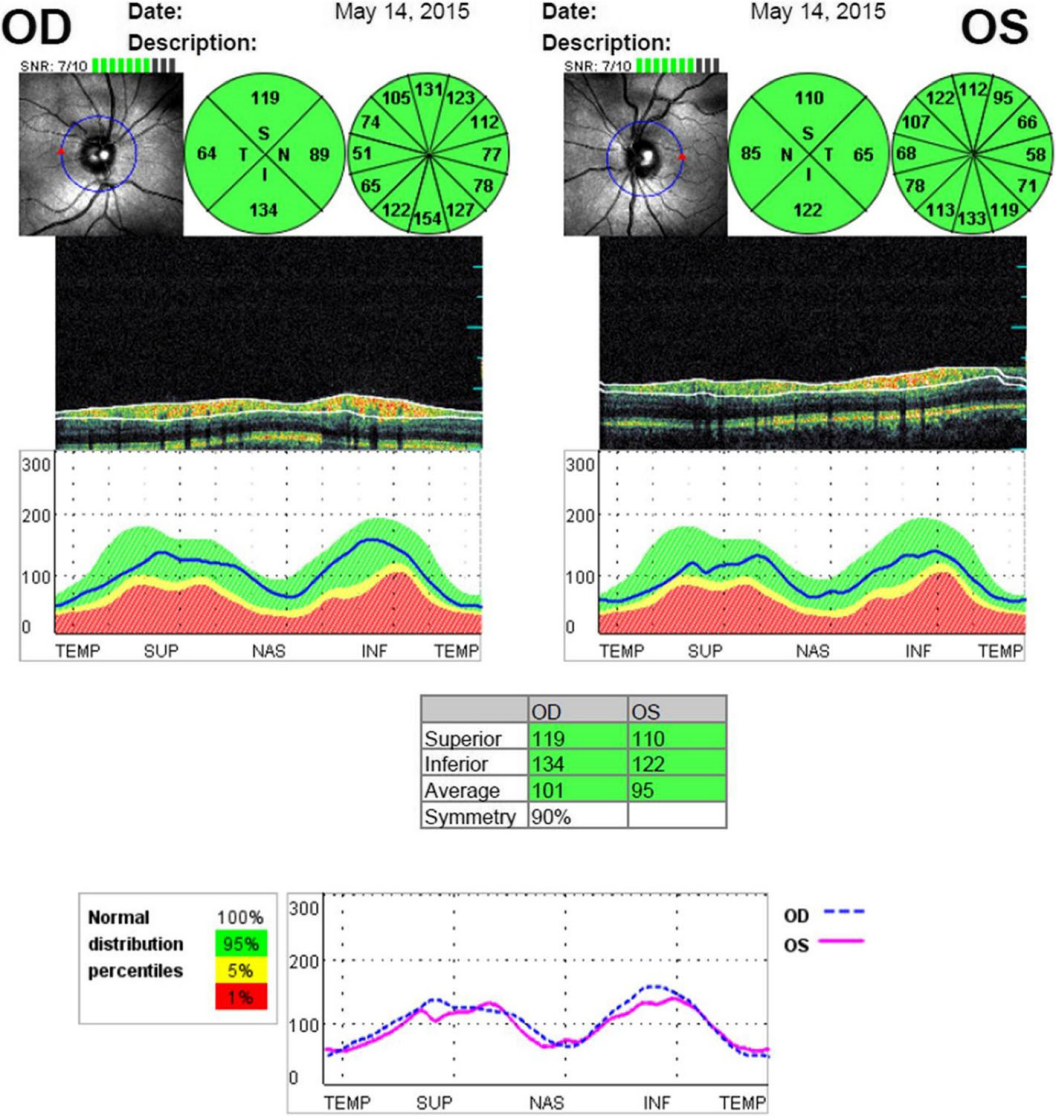
Peripapillary RNFL analysis with OCT in a patient with FMF. The RNFL thickness (inferior, superior, nasal, temporal quadrants and average) is measured around a 3.4 mm diameter circle centered on the optic disc

The FMF patients (21 males and 45 females) were divided into two main groups: 25 patients who had not used colchicine (untreated patient group) and 41 patients already using colchicine for more than 6 months (treated patient group). The control group consisted of 32 healthy subjects. The mean age was 32.82 ± 9.85 years in the untreated patient group, 36.53 ± 10.98 years in the treated patient group and 34.72 ± 9.07 years in the control group (*p* = 0.528). There was no statistically significant difference in gender (*p* = 0.983) or mean pRNFL thickness between the untreated patient group, treated patient group and healthy control group (*p* = 0.231). Similarly, we did not find a statistically significant difference between the three groups in pRNFL thickness in any quadrant (superior, inferior, temporal and nasal) (*p* = 0.583, *p* = 0.418, *p* = 0.817 and *p* = 0.081, respectively). The demographic characteristics are presented together with the pRNFL thickness for the study groups in Table 1. pRNFL OCT scan in a patient with FMF is shown in Fig. 1.

**Table 1:** Demographic characteristics and peripapillary RNFL thickness of the groups

Variables	Healthy Control Group (*n* = 32)	Untreated FMF Group (*n* = 25)	Treated FMF Group (*n* = 41)	Kruskal-Wallis test
Age (years) (mean ± SD)	34.72 ± 9.07	32.82 ± 9.85	36.53 ± 10.98	*p* = 0.528
Gender (male/female)	10/22	8/17	13/28	*p* = 0.983
Mean RNFL (μm) (mean ± SD)	98.18 ± 1.89	98.17 ± 7.32	101.41 ± 8.44	*p* = 0.231
Superior RNFL (μm) (mean ± SD)	117.04 ± 2.21	115.52 ± 10.28	115.75 ± 10.00	*p* = 0.583
Inferior RNFL (μm) (mean ± SD)	123.72 ± 2.84	125.88 ± 13.77	128.04 ± 12.14	*p* = 0.418
Temporal RNFL (μm) (mean ± SD)	70.45 ± 6.32	72.23 ± 12.16	72.85 ± 10.83	*p* = 0.817
Nasal RNFL (μm) (mean ± SD)	81.27 ± 4.16	80.47 ± 11.53	86.12 ± 12.27	*p* = 0.081

*FMF* familial Mediterranean fever, *RNFL* retinal nerve fiber layer, *μm* micrometer

Treated FMF patients were divided into two groups (6–24 months and longer than 24 months) according to the duration of colchicine use and compared to the control group and untreated patient group. No statistically significant difference in age and gender was found between these groups (*p* = 0.729, *p* = 0.995, respectively). The pRNFL thickness in each of the four quadrants and the mean value were also similar between the groups (all *p* > 0.05). These values are summarized together with demographic data in Table 2.

**Table 2:** Demographic characteristics and peripapillary RNFL thickness of the FMF and healthy control groups by colchicine use duration

Variables	Healthy Control Group (*n* = 32)	Untreated FMF Group (*n* = 25)	6–24 months Treated Group (*n* = 23)	> 24 months Treated Group (*n* = 18)	Kruskal-Wallis test
Age (years) (mean ± SD)	34.72 ± 9.07	32.82 ± 9.85	36.82 ± 11.48	36.16 ± 10.63	*p* = 0.729
Gender (male/female)	10/22	8/17	7/16	6/12	*p* = 0.995
Mean RNFL (μm) (mean ± SD)	98.18 ± 1.89	98.17 ± 7.32	100.95 ± 7.76	102.00 ± 9.44	*p* = 0.344
Superior RNFL (μm) (mean ± SD)	117.04 ± 2.21	115.52 ± 10.28	117.13 ± 10.45	114.00 ± 9.39	*p* = 0.596
Inferior RNFL (μm) (mean ± SD)	123.72 ± 2.84	125.88 ± 13.77	125.47 ± 9.76	131.33 ± 14.24	*p* = 0.312
Temporal RNFL (μm) (mean ± SD)	70.45 ± 6.32	72.23 ± 12.16	72.69 ± 11.00	73.05 ± 10.92	*p* = 0.899
Nasal RNFL (μm) (mean ± SD)	81.27 ± 4.16	80.47 ± 11.53	85.73 ± 13.85	86.61 ± 10.26	*p* = 0.106

*FMF* familial Mediterranean fever, *RNFL* retinal nerve fiber layer, *μm* micrometer

Treated FMF patients were divided into two groups according to the colchicine dose (< 1.5 mg/day and ≥1.5 mg/day) and were compared to the control group. We did not find a statistically significant difference between these groups for demographic data and the pRNFL thickness in the four quadrants and the mean value (Table 3).

**Table 3:** Demographic characteristics and peripapillary RNFL thickness of the FMF and healthy control groups by colchicine dosage

Variables	Healthy Control Group (n = 32)	< 1.5 mg/day (*n* = 21)	≥ 1.5 mg/day (*n* = 20)	Kruskal-Wallis test
Age (years) (mean ± SD)	34.72 ± 9.07	37.66 ± 11.67	35.35 ± 10.38	*p* = 0.746
Gender (male/female)	10/22	5/16	8/12	*p* = 0.543
Mean RNFL (μm) (mean ± SD)	98.18 ± 1.89	102.90 ± 7.32	99.85 ± 9.42	*p* = 0.150
Superior RNFL (μm) (mean ± SD)	117.04 ± 2.21	115.23 ± 8.96	116.30 ± 11.20	*p* = 0.405
Inferior RNFL (μm) (mean ± SD)	123.72 ± 2.84	130.04 ± 11.52	125.95 ± 12.70	*p* = 0.148
Temporal RNFL (μm) (mean ± SD)	70.45 ± 6.32	74.52 ± 11.58	71.10 ± 9.97	*p* = 0.363
Nasal RNFL (μm) (mean ± SD)	81.27 ± 4.16	87.76 ± 10.22	84.40 ± 14.17	*p* = 0.145

*FMF* familial Mediterranean fever, *RNFL* retinal nerve fiber layer, *μm* micrometer

## Discussion

We compared mean and quadrant pRNFL thickness values between FMF patients and age- and sex-matched healthy control subjects. FMF patients were divided according to their colchicine use (newly-diagnosed patients who were planned to start colchicine and patients already using colchicine), duration of use and dose of use and pRNFL thicknesses were investigated. We did not find a statistically significant difference in the mean and four quadrant pRNFL thicknesses between the FMF patient subgroups and the healthy control group.

There are only a few studies in the literature on ocular involvement in FMF. Yazici et al. [18] reported a series of 6 FMF patients; 2 with posterior uveitis, 2 with anterior uveitis, 1 with posterior scleritis and 1 with intermediate uveitis. Other reported ocular problems encountered in FMF patients include optic disc edema [19], amaurosis fugax [20], episcleritis [21], panuveitis [22], and ocular surface and tear-film abnormalities [23]. One of our patients had Behcet’s disease together with FMF and we observed hypopyon uveitis and during his follow-up. This patient was later excluded from the study.

Retinal and choroidal thickness in FMF patients has been investigated in a few other studies. Erdurmuş et al. [11] found no significant difference between FMF patients and controls when they measured retinal and choroidal thickness with OCT in pediatric FMF cases. They have suggested that the measurements were performed during the remission period in most patients and it is possible that taking measurements during an attack will alter the results. Gundogan et al. [12] found higher choroidal thickness values in FMF patients during an attack compared to the control group, suggesting a possible increase in choroidal thickness with increased inflammatory reaction in these patients. In our study we did not perform choroidal thickness measurements since choroidal thickness varies according to the vascular structure and may differ between acute attack and remission periods in FMF patients. We believe pRNFL thickness measurements with OCT would less like to be affected by such changes. Most of the newly-diagnosed patients we included in our study had recently experienced an acute attack. The lack of difference between the pRNFL values of the untreated patient group and treated patient group in the remission period in our study also supports that pRNFL values are not likely to be affected by the acute attack and remission periods.

A toxicity evaluation is recommended every 6 months in FMF patients using colchicine and we therefore did not include patients who had been using colchicine for less than 6 months [24]. No difference was found between pRNFL thicknesses of the groups receiving colchicine for 6–24 months and longer than 24 months. The classification of the patients based on the colchicine dose was made by considering that the effective adult dose of colchicine is reported as 1.5 mg/day [24]. No difference was found between pRNFL thickness of the subjects using a colchicine dose of 1.5 mg/day and those using 1.5 mg/day or more. According to these results, colchicine was found to have no dose- or duration-dependent effect on pRNFL thickness.

Colchicine is currently the gold standard for preventing FMF attacks and thus decreasing amyloidosis risk thanks to its anti-inflammatory effects. These effects are believed to be the result of leukocyte downstream function and microtubule disruption [25]. Animal studies have been conducted to investigate the effect of colchicine on ocular growth and retinal ganglion cells. Colchicine administered intravitreally to chickens was reported to damage the retinal ganglion cells and amacrine cells and induce ocular enlargement as a result [15, 16]. Besides, Leibovitch et al. [26] found colchicine in the tears of patients using systemic colchicine. We aimed to evaluate the possible effects of oral colchicine on pRNFL thickness as colchicine prevents FMF attacks, colchicine administered intravitreally is toxic to retinal structures, and oral colchicine can alter the ocular surface. Oral colchicine and FMF disease were seen to have no effect on pRNFL thickness. There is either no mention of the patients’ colchicine use in studies on pRNFL and choroid thickness measurements of FMF patients or all the OCT measurements were taken when the patients were on colchicine [10–12]. None of these studies on retinal and pRNFL thickness have reported any difference between FMF patients and the control group. It has been observed that choroidal thickness is increased in patients with FMF only during acute attack periods [12]. Alim et al. [13] investigated pRNFL and GCIPL (ganglion cell-inner plexiform layer) thicknesses of adult-onset FMF patients in terms of DSS (disease severity score), daily colchicine dosage, colchicine use duration and annual number of FMF attacks and found no significant difference between the groups. In contrast, we only evaluated pRNFL thicknesses of newly-diagnosed FMF patients who had not been started colchicine and patients on colchicine treatment in our study. No difference was found between the control group, untreated patient group and treated patient group in terms of pRNFL thickness.

Limitations to this study include being a single-center study, the small size of the FMF patient groups, the inability to show association between patient’s blood samples of TNF-alpha/or IL-1β and pRNFL thickness, and the lack of pediatric patients. However, we believe that our study provides useful information about a rather less-studied clinical entity and that it will help guiding future studies with larger number patients from all age groups in shedding light on to ocular manifestations of FMF.

## Conclusion

To our knowledge, this is the first study to compare the pRNFL thickness of newly-diagnosed colchicine-naïve FMF patients and those on colchicine. Despite the low number of patients, our results suggest that oral colchicine has no effect on pRNFL thickness in FMF patients. Our study also suggests that duration of oral colchicine use and dose of oral colchicine had no effect on pRNFL thickness in FMF patients.

## Abbreviations

BCVA: Best-corrected visual acuity
FMF: Familial Mediterranean fever
GCIPL: Ganglion cell-inner plexiform layer
pRNFL: Peripapillary retinal nerve fiber layer
SD-OCT: Spectral-domain optical coherence tomography
Μm: Micrometer
